# Who Benefits From Humor-Based Positive Psychology Interventions? The Moderating Effects of Personality Traits and Sense of Humor

**DOI:** 10.3389/fpsyg.2018.00821

**Published:** 2018-05-28

**Authors:** Sara Wellenzohn, René T. Proyer, Willibald Ruch

**Affiliations:** ^1^Department of Psychology, University of Zurich, Zurich, Switzerland; ^2^Department of Psychology, Martin Luther University of Halle-Wittenberg, Halle, Germany

**Keywords:** happiness, humor, personality, positive psychology, positive psychology interventions

## Abstract

The evidence for the effectiveness of humor-based positive psychology interventions (PPIs; i.e., interventions aimed at enhancing happiness and lowering depressive symptoms) is steadily increasing. However, little is known about who benefits most from them. We aim at narrowing this gap by examining whether personality traits and sense of humor moderate the long-term effects of humor-based interventions on happiness and depressive symptoms. We conducted two placebo-controlled online-intervention studies testing for moderation effects. In Study 1 (*N* = 104) we tested for moderation effects of basic personality traits (i.e., psychoticism, extraversion, and neuroticism) in the three funny things intervention, a humor-based PPI. In Study 2 (*N* = 632) we tested for moderation effects of the sense of humor in five different humor-based interventions. Happiness and depressive symptoms were assessed before and after the intervention, as well as after 1, 3, and 6 months. In Study 2, we assessed sense of humor before and 1 month after the intervention to investigate if changes in sense of humor go along with changes in happiness and depressive symptoms. We found moderating effects only for extraversion. Extraverts benefitted more from the three funny things intervention than introverts. For neuroticism and psychoticism no moderation effects were found. For sense of humor, no moderating effects were found for the effectiveness of the five humor-based interventions tested in Study 2. However, changes in sense of humor from pretest to the 1-month follow-up predicted changes in happiness and depressive symptoms. Taking a closer look, the playful attitude- and sense of humor-subscales predicted changes in happiness and depression for up to 6 months. Overall, moderating effects for personality (i.e., extraversion) were found, but none for sense of humor at baseline. However, increases in sense of humor during and after the intervention were associated with the interventions’ effectiveness. Thus, we found humor-based interventions to be equally suited for humorous and non-humorous people, but increases in the sense of humor during the intervention phase could serve as an indicator whether it is worth continuing the intervention in the long-term.

## Introduction

Positive Psychology is the scientific study of what makes life most worth living (Seligman and Csikszentmihalyi, 2000). It aims at promoting psychological research and practice in areas such as morally positively valued traits (character strengths), positive emotions, and positive institutions and their contribution to well-being. Another core topic of positive psychology is the development of so-called positive psychology interventions (PPIs; i.e., “[…] treatment methods or intentional activities that aim to cultivate positive feelings, behaviors, or cognitions"; Sin and Lyubomirsky, 2009, p. 468). Recent meta-analyses by Sin and Lyubomirsky (2009) and Bolier et al. (2013) found support for the notion that they are effective in enhancing happiness and ameliorating depressive symptoms.

One specific variant of PPIs are interventions, which focus on humor. Previous research provides support for the notion that they can enhance well-being in the general population (e.g., McGhee, 2010b; Crawford and Caltabiano, 2011; Gander et al., 2013; Proyer et al., 2014; Wellenzohn et al., 2016b; for an overview see Ruch and McGhee, 2014; Ruch and Hofmann, 2017), but also in clinical samples [e.g., Hirsch et al., 2010; Falkenberg et al., 2011; Konradt et al., 2013; see also Berger et al. (2017)]. There are group-administered training programs for humor that were found to be effective for enhancing emotional well-being, life satisfaction, psychological well-being, subjective health, positive mood, optimism, and lowering depression, feelings of stress or suicidal tendencies (e.g., Papousek and Schulter, 2008; Hirsch et al., 2010; Crawford and Caltabiano, 2011; Falkenberg et al., 2011; Ruch et al., 2018b; Tagalidou et al., 2018, Tagalidou et al., in press; for an overview see McGhee, 2010a,b). Thus, humor-based PPIs are expected to be well-received by the participants and enable a higher commitment to continue practicing and incorporating the activities into daily life. It has been shown that humor induces amusement (Ruch, 2001, 2008, 2009; Auerbach et al., 2016), an important facet of positive emotions (the one that most frequently goes along with laughter; Platt et al., 2013). Given that the elicitation of positive emotions is one of the proposed working mechanisms of PPIs (Sin and Lyubomirsky, 2009), humor seems to be particularly well-suited for incorporation in PPIs. Furthermore, Wellenzohn et al. (2016a) found support for savoring positive emotions serving as a working mechanism in humor-based PPIs.

While evidence for the effectiveness of PPIs is steadily growing, only little knowledge exists on whether (and how) certain personality traits moderate these effects. This is especially of interest from an applied perspective since the person × intervention fit (i.e., the degree to which an intervention matches an individual’s preferences and personality) is associated with an intervention’s effectiveness (e.g., Schueller, 2010, 2012, 2014; Proyer et al., 2015). We report two studies that are aimed at narrowing this gap in the literature by testing the impact of basic personality traits and sense of humor as defined by McGhee (1999, 2010a) as moderators in humor-based PPIs.

### Humor-Based Online Positive Psychology Interventions

Seligman et al. (2005) published the first large-scale online placebo-controlled PPI study. They report findings for three self-administered online PPIs that are effective for up to 6 months in ameliorating depressive symptoms and enhancing happiness in comparison with a placebo control condition: The *gratitude visit-* (i.e., writing and delivering a gratitude letter to a person who has not been thanked so far), *three good things-* (i.e., writing down three good things that happened during the day), and *using signature strengths in a new way*-intervention (i.e., participants complete a character strengths inventory and receive feedback on their five highest strengths and the instruction to apply these strengths in a new way). An advantage of these online programs is that they are more cost effective than programs in group- or individual-settings as they are scalable (i.e., they can be easily distributed and made accessible to a large number of interested users) and can be self-administered using standardized written instructions; both are typically associated with low expenses for the researcher applying and supervising these programs in practice. There is also initial experience with humor-based online interventions. For example, Gander et al. (2013) adapted the *three good things*-intervention to a *three-funny things*-intervention by changing the instruction to include humor as its core component—instead of writing down three good things that happened to the person during the day, participants were asked to write down three *funny* things that happened to them during the day. The authors found the intervention to be effective in enhancing happiness for up to 3 months and ameliorating depressive symptoms up to 6 months after the intervention-week compared to a placebo control condition. Similar effects were recently found for a sample of people aged 50–79 years (Proyer et al., 2014).

A third study by Wellenzohn et al. (2016b) replicated the findings for the *three funny things*-intervention and adapted four other well-established PPIs into 1-week humor-based PPIs (see Wellenzohn et al., 2016b for a more detailed description of the interventions); namely, (a) the *gratitude visit*- (Seligman et al., 2005) was adapted into the *collecting funny things*-intervention (i.e., remembering the funniest things ever experienced and writing them down in as much detail as possible); (b) the *counting kindness*- (Otake et al., 2006) into the *counting funny things*-intervention (i.e., counting all funny things that happen during the day and note the total number); (c) the *using your signature strengths in a new way*- (Seligman et al., 2005) into the *applying humor-*intervention (i.e., noticing the humorous experiences during the day and add humorous activities); and (d) the *one door closes and another door opens*- (Rashid and Anjum, 2008) into the *solving stressful situations in a humorous way*-intervention (i.e., thinking about a stressful experience and how it could have been solved in a humorous way). These newly adapted interventions (self-administered over 1 week) were then tested in an online-setting by comparing their long-term effectiveness with a placebo control condition (early childhood memories as in Seligman et al., 2005). As in earlier studies, the *three funny things*-intervention was effective in increasing well-being, but there were no effects for depression. Furthermore, two out of the four newly adapted humor-based PPIs enhanced happiness (counting funny things- and applying humor-) and two were effective in ameliorating depressive symptoms (*applying humor*- and *solving stressful situations in a humorous way*-intervention) for up to 6 months. Hence, three out of the five tested interventions were effective in enhancing well-being and ameliorating depression and more research in this area seems warranted.

### Who Benefits Most From a Humor-Based Positive Psychology Intervention?

Thus far, only few studies have directly examined the influence of individual difference variables in PPIs, and the findings are mixed. Senf and Liau (2013) showed that higher levels in extraversion and openness contribute to greater increases in happiness after a gratitude-based intervention. Greater extraversion was also associated with a stronger reduction in depressive symptoms following a gratitude- and a strengths-based intervention. Schueller (2012) also found that extraverted participants benefit more from a gratitude-intervention, as well as from a savoring-intervention. However, contrary to the findings by Senf and Liau (2013), Schueller found stronger benefits for introverts from a strengths-based-intervention. Furthermore, he also found introverts to benefit more from an *active-constructive responding-* and a *three good things*-intervention. Extraversion seems to play an important role for the effectiveness of interventions (e.g., when having to interact with others or share experiences with others), this could also be expected by extensive literature that supports robust positive associations of extraversion with well-being (e.g., Pavot et al., 1990; Oerlemans and Bakker, 2014). Ng (2015) tested the role of neuroticism in a gratitude/kindness-intervention and found that participants with low levels in neuroticism demonstrated greater increases in happiness. However, a recent study using a randomized, group-based-design for interventions targeting the components of Seligman’s (2002) Authentic Happiness Theory (i.e., the pleasurable, engaged, and meaningful life) has found no moderating effect of personality in the sense of the big five personality traits (Proyer et al., 2016). In the same line, Wang et al. (2017) did not find any moderating effects of personality for a well-being intervention in adolescents (only for the control phase). Hence, several studies suggest that individual difference variables moderate the effectiveness of some PPIs and encourage further research into the person × intervention fit as there seem to be intervention-specific differences in how far personality variables may have an impact. Thus far, no study has tested moderating effects of individual differences variables in humor-based interventions. Based on the existing literature, we expect humor-based PPIs to work better for those higher in extraversion. This hypothesis also receives support from correlational studies showing a positive relation between measures of humor and extraversion (e.g., Köhler and Ruch, 1996).

In addition to basic personality traits, *sense of humor* might be an important moderating variable for humor-based interventions. There are numerous conceptualizations of the sense of humor (for an overview see Ruch, 2007, 2008). McGhee (1999) provides a multi-faceted model that is based on six hierarchically ordered humor-skills or -habits (i.e., enjoyment of humor, laughter, verbal humor, humor in everyday life, laughing at oneself and finding humor under stress). He argues that these humor-skills are malleable in order to increase ones sense of humor (McGhee, 2010a,b). McGhee defines sense of humor as an ability to cope with stressful situations in daily life. He sees playfulness as its basis and argues that humor is a variant of play, namely the play with ideas (for an overview see Ruch and Heintz, 2018). A playful attitude can be seen as a facilitating frame of mind for establishing humor and for successfully processing humorous stimuli along with positive mood. McGhee’s (1999) framework seems best-suited for a further exploration in PPI studies as he also developed a measure specifically for usage in intervention studies (i.e., the *Sense of Humor Scale;* McGhee, 2010a). We aim to test Wellenzohn et al.’s (2016b) hypothesis on the moderating role of the sense of humor in humor-based PPIs and its potential in predicting long-term changes in happiness and depressive symptoms.

### The Present Studies

Our main aim is to examine the moderating effects of personality and the sense of humor on the effectiveness of humor-based interventions in a set of two studies. In Study 1, we test basic personality traits (i.e., the superfactors of personality *psychoticism, extraversion*, and *neuroticism* in Eysenck’s personality model; see e.g., Eysenck and Eysenck, 1985) as moderators for the effectiveness of the *three funny things*-intervention (re-analyzing data from the study by Gander et al., 2013). Based on the existing literature, we expect humor-based PPIs to be more effective for people low in neuroticism and high in extraversion. In Study 2, we examine sense of humor as conceptualized by McGhee (2010a) as a moderator in the *three funny things*-intervention as well as in four further humor-based PPIs (re-analyzing data from the study by Wellenzohn et al., 2016b). Furthermore, we test (a) whether changes in sense of humor from pretest to the 1-month follow-up can predict long-term changes in happiness and depressive symptoms, and (b) whether changes in sense of humor and its sub-components differ in their ability to predict changes in happiness and depressive symptoms. Both studies are placebo-controlled online intervention-studies with happiness and depressive symptoms assessed at pre- and posttest as well as at 1, 3, and 6 months follow-ups.

Those with a higher sense of humor (according to McGhee’s conceptualization; McGhee, 2010a) are more often exposed to humorous situations and thus, might come up with funny things to write down more easily (the core of the three funny things-intervention), to remember (as in the collecting funny things-intervention), or also noticing funny things during the day more easily (as in the counting funny things-intervention). Moreover, those with high scores in sense of humor might also find it easier to come up with ideas on how and where to apply humor in a new way (as in the applying humor-intervention), or be more creative in solving stressful situations in a humorous way. Thus, we expect those with higher levels in sense of humor to benefit more from humor-based PPIs. Furthermore, as the sense of humor might be a trigger of positive emotions, we expect early changes in sense of humor and its sub-components to predict upward changes in happiness and amelioration of depression.

## Study 1

### Method

#### Participants

The total sample consisted of *N* = 104 women who completed all follow-up assignments in the *three funny things*-intervention (*n* = 55) or the placebo control condition (*n* = 49) in the study^1^ by Gander et al. (2013). Their mean age was 45.16 years (*SD* = 9.75), ranging from 19 to 79. The participants were generally well-educated, with 26.9% having a university degree, 17.3% having a degree from an applied university, 22.1% having a certificate that would allow them to attend university, and 33.7% having completed vocational training.

#### Instruments

The *Eysenck Personality Questionnaire-Revised* (EPQ-R; Eysenck and Eysenck, 1985; German version by Ruch, 1999) consists of 102 items with a yes/no answer-format for the assessment of psychoticism (32 items, α = 0.63), extraversion (23 items, α = 0.79), and neuroticism (25 items, α = 0.84), and additionally a lie scale (22 items, α = 0.74) to cover social desirability.

The *Authentic Happiness Inventory* (AHI; Seligman et al., 2005) is a subjective measure for the assessment of overall happiness in the past week. Its reliability and validity, in the original as well as the German version, was supported by a broad range of studies (e.g., Ruch et al., 2010; Proyer et al., 2015). Every item consists of five statements (e.g., from to “Most of the time I feel bored” to “Most of the time I feel fascinated by what I am doing”). In Study 1, a 33-item version was used and in Study 2 a newer, revised version with 24 items was used. Internal consistency at pretest in Study 1 was α = 0.91.

The *Center for Epidemiologic Studies Depression Scale* (CES-D; Radloff, 1977; in the German Adaption by Hautzinger and Bailer, 1993) consists of 20-items with a four-point scale ranging from 0 (*Rarely or none of the time [Less than 1 day]*) to 3 (*Most or all of the time [5–7 days]*) and measures the frequency of depressive symptoms in the past week (e.g., “My sleep was restless”). Internal consistency at pretest in Study 1 was α = 0.92.

#### Procedure

The study was advertised as a free strengths-training in leaflets, in newspapers and magazines. The participants registered on a website that was set up for the administration of the program and were randomly assigned to either the *three funny things*-intervention (i.e., writing down three funny things that happened during the day), or the placebo control condition (i.e., writing about early childhood memories; see Seligman et al., 2005; Gander et al., 2013). All participants filled in the basic demographics and baseline-questionnaires (i.e., AHI, CES-D, and EPQ-R). They subsequently received instructions for the intervention and conducted the intervention for the following seven consecutive days. After the intervention-week, as well as 1, 3, and 6 months after the intervention, they logged on to the website and completed the AHI and the CES-D. Participants received an automatically generated personalized feedback on their well-being scores over the course of 6 months at the end of the study.

### Results

#### Preliminary Analysis

Descriptive statistics for the AHI (*M* = 2.98, *SD* = 0.49), the CES-D (*M* = 15.56, *SD* = 10.73) and the EPQ-R as well as correlations between the personality variables and the AHI and CES-D at pretest are presented in **Table 1**. The table shows the expected findings in the cross-sectional analysis. Extraversion was robustly positively correlated with happiness and negatively with depression, while neuroticism demonstrated a negative relation with happiness, but was positively associated with depression.

**Table 1:** Descriptive statistics and moderating effects of personality at baseline on happiness and depressive symptoms in the three funny things condition compared to the placebo control-condition for Study 1.

			AHI	CES-D	Happiness					Depression				
	*M*	*SD*	*r*	*r*	*df*	*B*	*SE B*	*t*	*p*	*df*	*B*	*SE B*	*t*	*p*
Psychoticism	7.68	3.42	-0.02	-0.06	99	-13.67	18.60	-0.74	0.23	99	11.97	9.68	1.24	0.11
Extraversion	11.96	4.46	0.39***	-0.20*	99	23.46	9.88	2.37	0.01	99	-9.19	5.24	-1.75	0.04
Neuroticism	14.00	5.25	-0.53***	0.41***	99	-5.84	9.41	-0.62	0.27	99	0.81	4.73	0.17	0.43
Lie-scale	8.36	3.81	-0.05	0.08	99	-0.34	11.74	-0.03	0.49	99	-1.50	6.12	-0.24	0.41

*N = 104. r = partial correlation with AHI/CES-D at pretest controlled for age. Happiness/Depression = Personality × condition interaction (0 = Placebo control condition, 1 = Three funny things-intervention) as predictor of the happiness/depression scores after the intervention (all follow-ups averaged), when controlling for pretest scores in happiness/depression and personality. ^∗^p < 0.05, ^∗∗^p < 0.01, ^∗∗∗^p < 0.001 (two-tailed)*.

#### Moderating Effects of Personality

**FIGURE 1:**
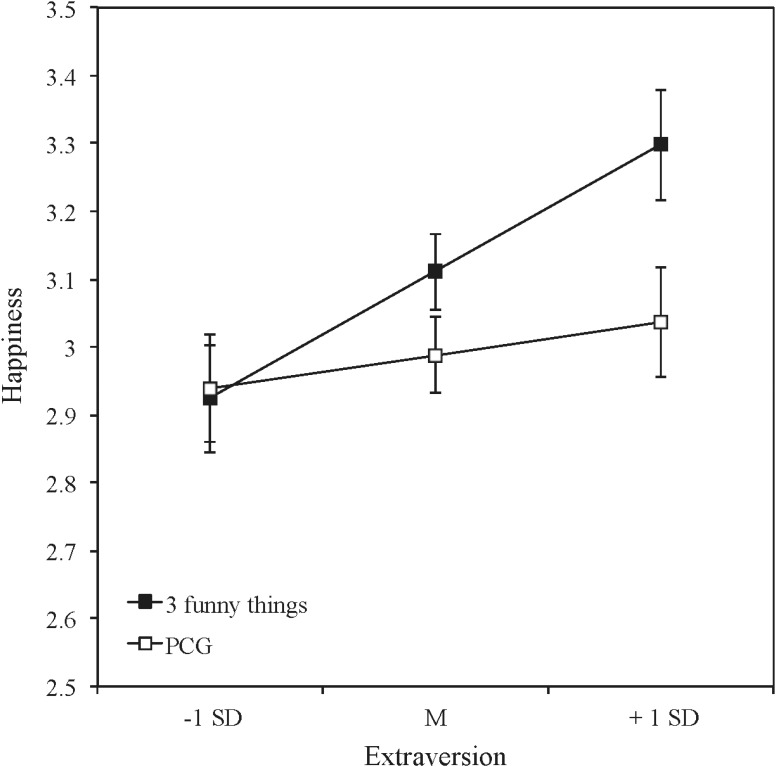
Happiness after the intervention (all time periods averaged, controlled for the pretest scores) for the three funny things condition and the placebo control condition (PCG) for different levels of extraversion.

**FIGURE 2:**
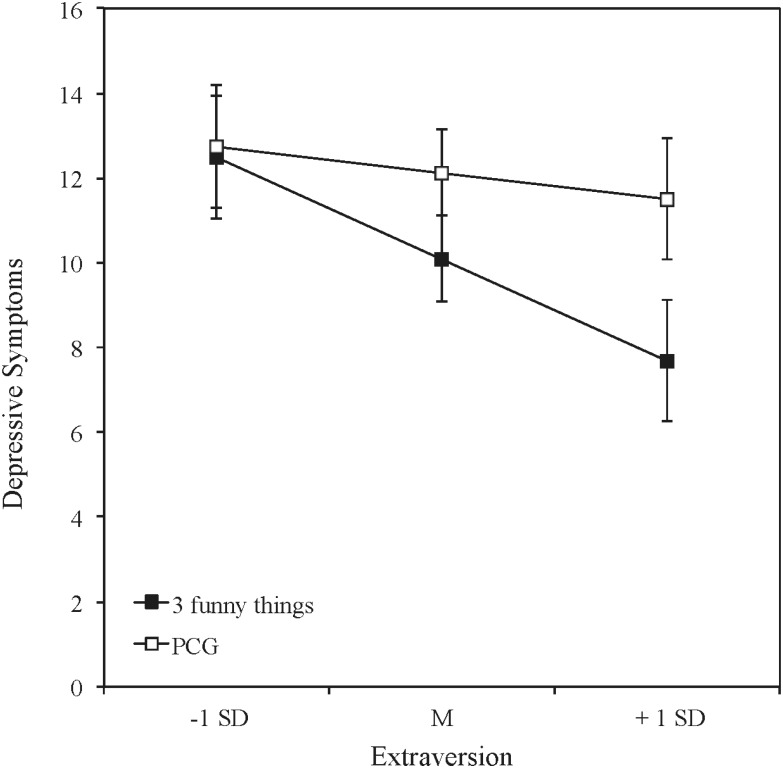
Depressive symptoms after the intervention (all time periods averaged, controlled for the pretest scores) for the three funny things condition and the placebo control condition (PCG) for different levels of extraversion.

In order to test potential moderating effects of the three personality dimensions (extraversion, neuroticism, and psychoticism), we computed hierarchical regression analyses. We analyzed interaction effects between each personality dimension and the group-condition on happiness (averaged over the four follow-ups), controlling for the baseline level in the AHI. For this calculation we used the SPSS PROCESS-macro by Hayes (2013). This macro allows analyzing the direct effect (regression controlled for the mediator), the total effect (regression without including the mediator), and the indirect effect. The same analyses were conducted for depressive symptoms (see **Table 1** for the interaction effects). Extraversion moderated the effectiveness of the intervention on happiness and also on depressive symptoms. **Figures 1**, **2** show the direction of the interaction-effects of extraversion for happiness and depressive symptoms. Error Bars represent the standard errors of the group differences.

Higher levels in extraversion went along with greater increases in happiness (**Figure 1**) and greater decreases in depressive symptoms (**Figure 2**) in the *three funny things*-intervention in comparison with the placebo control condition.

While Study 1 has shown that extraversion plays a role for the effectiveness of a humor-based PPI, Study 2 examines the role of individual differences in the sense of humor as an additional moderator in PPIs.

## Study 2

### Method

#### Participants

Of the 1,472 participants who have started the intervention (thereof, 243 in the placebo control condition), we used a sample of *N* = 632 adults (117 men and 515 women) who completed all follow-up measurements in the study by Wellenzohn et al. (2016b). The participants’ mean age was 47.38 (*SD* = 11.55) and they were rather well educated with 41.5% having a university degree, 19.1% having a degree from an applied university, 22.1% having a certificate that would allow them to attend university, and 3.5% having completed vocational training.

#### Instruments

As in Study 1, the AHI (α = 0.93) and the CES-D (α = 0.88) were used.

The *Sense of Humor Scale* (SHS: by McGhee, 2010a; used in the German version by Proyer et al., 2010) assesses *playfulness* vs. *serious attitude*, *positive* vs. *negative mood* and *sense of humor* with its six sub-facets (enjoyment of humor, laughter, verbal humor, humor in everyday life, laughing at yourself, and humor under stress), as well as a total score for a more global assessment of sense of humor (see Müller and Ruch, 2011; Ruch and Heintz, 2018). The internal consistency at pre-test was α = 0.92 for the SHS Total Score, α = 0.71 for the playfulness dimension, α = 0.85 for the mood dimension, and α = 0.85 for sense of humor (for its sub-facets it ranged from α = 0.51 for the enjoyment of humor to α = 0.84 for the humor under stress sub-facet; median = 0.69). The SHS consists of 40 items (e.g., “I often find humor in things that happen at work”) on a 7-point answer-scale.

#### Procedure

The procedure is comparable to Study 1 using the same recruitment strategy, but data were collected independently in the two studies. Participants were randomly assigned to one of the five humor-based PPIs (short descriptions are given in the introduction of the present article) or the placebo control-condition (i.e., writing about early childhood experiences). The dropout rate in the intervention groups varied between 55.3% and 58.3%, and was 56.8% for the placebo control condition. Happiness and depressive symptoms were also assessed at pre- and posttest as well as at follow-up after 1-, 3-, and 6-months. Participants completed the SHS at pretest and at the 1-month follow-up.

### Results

#### Preliminary Analyses

Descriptive statistics and the relations between the SHS scales and the AHI and CES-D at pretest are presented in **Table 2**.

**Table 2:** Bivariate correlations between the AHI, the CES-D, and the components of the sense of humor scale controlled for age and sex for Study 2.

	*M*	*SD*	*r*_AHI_	*r*_CES-D_
AHI at pretest	3.16	0.48		
CES-D at pretest	10.28	5.70		
SHS tot	4.47	0.71	0.51	-0.38
Playful	4.86	0.80	0.45	-0.33
Mood	4.81	0.99	0.69	-0.56
SoH	4.23	0.80	0.32	-0.23
Enjoy	3.79	0.98	0.11	-0.05
Laughter	3.75	1.04	0.31	-0.24
Verbal	3.99	1.05	0.24	-0.19
Eday	4.92	0.94	0.33	-0.25
YSelf	4.66	1.10	0.22	-0.15
Stress	4.28	1.21	0.25	-0.15

*N = 628. AHI, Authentic Happiness Inventory; CES-D, Center for Epidemiologic Studies Depression Scale; SHS tot, total score in the Sense of Humor Scale; Playful, playful vs. serious attitude; Mood, positive vs. negative mood; SoH, sense of humor; Enjoy, enjoyment of humor; Verbal, verbal humor; Eday, humor in everyday life; YSelf, laughing at yourself; Stress, humor under stress. All correlations are significant at the 0.1%-level (two-tailed) except for “enjoy humor” at 1% for the AHI and non-significant for the CES-D.*

The table shows that the means are in the expected range. Correlations with happiness and depressive symptoms were comparable with those reported by Proyer et al. (2010) for personal well-being. The dependent variables were robustly negatively correlated at pretest (*r* = -0.58, *p* < 0.01) without indicating redundancy.

#### Moderating Effects of Sense of Humor

To examine the moderating role of the sense of humor as measured with the SHS (McGhee, 2010a) on the effectiveness of humor-based PPIs, we computed the interaction-effects between the conditions (i.e., the humor-based PPIs vs. the placebo control condition) and the SHS Total Score on happiness and depressive symptoms, averaged over the four follow-ups, while controlling for pretest scores in happiness and depressive symptoms, and the SHS Total Score. As in Study 1, the same macro by Hayes (2013) was used for the analyses (**Table 3**).

**Table 3:** Moderating effects of the sense of humor total score at baseline on happiness and depressive symptoms in five different humor-based interventions compared to the placebo control-condition (*n* = 105) for Study 2.

	*n*	Happiness					Depression				
		*df*	*B*	*SE B*	*t*	*p*	*df*	*B*	*SE B*	*t*	*p*
Three funny things	101	201	-0.04	0.06	-0.70	0.48	201	0.05	0.05	0.96	0.34
Collecting funny things	105	205	-0.00	0.05	-0.02	0.98	205	0.02	0.05	0.32	0.74
Counting funny things	108	208	0.01	0.06	0.11	0.92	208	0.03	0.05	0.62	0.54
Applying humor	104	204	-0.02	0.06	-0.32	0.74	204	-0.00	0.05	-0.02	0.98
Solving stressful situations	109	209	-0.03	0.06	-0.49	0.62	209	0.07	0.05	1.40	0.16

*Happiness/Depression = Sense of humor × condition interaction (0 = Placebo control condition, 1 = Humor-based intervention) as predictor of the happiness/depression scores after the intervention (all follow-ups averaged), when controlling for pretest scores in happiness/depression and sense of humor. Solving stressful situations = Solving stressful situations in a humorous way. *p* (*two-tailed*)*.

**Table 3** shows that *none* of the interaction-effects were significant.

While **Table 3** shows the analyses for the total score of the SHS only, we also computed the respective analyses for the playfulness scale, the positive vs. negative mood scale, the sense of humor scale, and the six humor skills. However, none of these analyses showed significant interaction effects (findings are not shown in detail, but are available upon request from the authors). In these analyses, the *t*-values for happiness ranged between 0.00 and 0.79 (median = 0.02) and between 0.02 and 1.40 (median = 0.15) for depression (all *n.s.*).

For a more in-depth analysis, initial changes in the SHS scales (changes from baseline to 1 month after completion of the intervention) were used for the prediction of changes in happiness and depressive symptoms (=criteria). Hierarchical regression analyses were conducted. In Step 1 age and sex were entered as predictors (yielding no incremental contribution in the prediction of happiness or depression; ≤0.01%). In Step 2, the initial changes in the SHS scales (changes from pretest to the 1-month follow-up) were entered as predictors of changes in happiness and depressive symptoms. The analyses were conducted for a total score of changes (i.e., an average score for the 1-, 3-, and 6-months follow-ups), but also separately for changes from the pretest to the 1 month follow-up, the 3 months follow-up, and the 6 months follow-up. The results for Step 2 are displayed in **Table 4**.

**Table 4:** Hierarchical regression analyses (step 2) of initial changes in sense of humor and its components on changes in happiness and depressive symptoms in the humor-based PPIs controlled for age and sex for Study 2.

		Changes in all follow-ups		Changes after 1 month		Changes after 3 months		Changes after 6 months	
Initial changes		Δ*F*	Δ*R*^2^	Δ*F*	Δ*R*^2^	Δ*F*	Δ*R*^2^	Δ*F*	Δ*R*^2^
SHS tot	AHI	113.81***	0.18	194.05***	0.27	60.11***	0.10	36.74***	0.07
	CES-D	35.08**	0.06	104.79***	0.17	9.67**	0.02	1.76	0.00
Playful	AHI	41.73***	0.07	53.27***	0.09	27.54***	0.05	16.59***	0.03
	CES-D	7.13**	0.01	22.02***	0.04	2.64	0.01	0.04	0.00
Mood	AHI	116.31***	0.18	243.69***	0.32	55.46***	0.10	31.31***	0.07
	CES-D	66.52***	0.11	211.03***	0.30	21.05***	0.04	2.77^†^	0.01
SoH	AHI	57.56***	0.10	86.57***	0.14	32.15***	0.06	21.52***	0.04
	CES-D	13.07***	0.02	36.74***	0.07	2.64	0.01	1.12	0.00
Enjoy	AHI	18.54***	0.03	23.97***	0.04	10.75**	0.02	8.58**	0.02
	CES-D	1.40	0.00	6.49*	0.01	0.03	0.00	0.22	0.00
Laughter	AHI	24.33***	0.04	39.58***	0.07	13.15***	0.02	8.44**	0.02
	CES-D	6.01*	0.01	19.53***	0.04	1.49	0.00	0.11	0.00
Verbal	AHI	27.40***	0.05	27.97***	0.05	23.98***	0.04	10.57**	0.02
	CES-D	3.45^†^	0.01	8.74**	0.02	1.20	0.00	0.17	0.00
Eday	AHI	25.40***	0.05	40.46***	0.07	14.55***	0.03	8.53**	0.02
	CES-D	9.23**	0.02	23.26***	0.04	2.86^†^	0.01	0.63	0.00
YSelf	AHI	21.44***	0.04	36.69***	0.07	7.59**	0.01	10.39**	0.02
	CES-D	8.87**	0.02	13.33***	0.03	0.62	0.00	5.97*	0.01
Stress	AHI	19.11***	0.04	30.56***	0.06	10.84**	0.02	6.46*	0.01
	CES-D	5.68*	0.01	20.83***	0.04	2.59	0.01	0.07	0.00


*N = 527. PPIs, positive psychology interventions ; Initial changes, changes in sense of humor and its components from pretest to the 1-month follow-up; AHI, Authentic Happiness Inventory; CES-D, Center for Epidemiologic Studies Depression Scale; SHS tot, total score in the Sense of Humor Scale; Playful, playful vs. serious attitude; Mood, positive vs. negative mood; SoH, sense of humor; Enjoy, enjoyment of humor; Verbal, verbal humor; Eday, humor in everyday life; YSelf, laughing at yourself; Stress, humor under stress; Changes in happiness = changes in happiness from pretest to the averaged follow-ups; Changes in depression = changes in depressive symptoms from pretest to the averaged follow-ups. ^†^p < 0.10, ^∗^p < 0.05, ^∗∗^p < 0.01, ^∗∗∗^p < 0.001 (two-tailed).*

The table shows that, as expected, early changes in humor predicted changes in happiness and in depressive symptoms at most of the time points. The multiple squared correlation coefficients for Step 2 for the averaged follow-ups ranged between 0.03 (enjoyment of humor) and 0.18 (total score of the SHS; median = 0.05) for happiness and between 0.00 (enjoyment of humor) and 0.11 (positive mood; median = 0.02) for depression. On average, these coefficients were larger for the 1-month follow-up than for the later follow-ups, but the trends were more or less comparable in all cases.

## Discussion

This study provides first data on moderating effects of three basic personality traits on a *humor*-based PPI; namely, the *three funny things*-intervention. Those higher in *extraversion* demonstrated greater benefit from the intervention. This finding is in line with data on positive associations of extraversion and well-being (e.g., Pavot et al., 1990; Oerlemans and Bakker, 2014). We did not find effects for psychoticism and neuroticism; also the tendency toward socially desirable answering behavior was not related to the interventions’ effectiveness. For psychoticism, the coefficients might have been slightly affected by the comparatively low reliability of this scale. The findings for extraversion are in line with Senf and Liau’s (2013) work, who found similar results for a signature strengths and gratitude intervention (see Seligman et al., 2005). Similarly, Schueller (2012) found, when varying the gratitude visit-intervention with different degrees of social interactions needed, that delivering a gratitude letter in person also yielded greater benefits for those higher in extraversion, than without any personal contact. One might argue that the *three funny things*-intervention (at least implicitly) also addresses social interaction situations—as funny things might be more likely to be experienced in the company of others or that people actively engaged in more contact with others for experiencing more funny things. The latter would be in line with findings that only behaving more extravert could already contribute to a persons’ well-being (see Fleeson et al., 2002).

It might be advisable to include variations of the standard instructions in future studies to make the activity more accessible to introverts. Otherwise, a different humor-based intervention (see McGhee, 1999, 2010a; Wellenzohn et al., 2016b; Ruch and Hofmann, 2017) may be more suitable for those low in extraversion. One might speculate that presenting ideas on situations or experience that provide humorous incidents without other people being present might make this intervention equally effective for extraverts and introverts. Hence, one aim for future application might be to develop interventions that are equally suitable for individuals with different levels of extraversion, or change the instructions in a way that all can work well with the included activities (e.g., introverts might find additional examples of observing humor in situations with people they know well rather than with strangers or persons that are less well-known to them, easier to work with).

Findings of Study 2 show that the sense of humor (as conceptualized by McGhee, 1999, 2010a) had *no* moderating effects on the effectiveness of five humor-based interventions. From a practical point of view this can be seen as “good news” since participants with varying levels of sense of humor (not only those with greater inclinations) seem to benefit from these interventions. It seems as if the interventions are accessible to participants similarly irrespective of self-reported sense of humor. Although there were some trends in the conducted analyses, they seem to be negligible from a practical point of view.

Although, McGhee’s (2010a) *Sense of Humor Scale* is only one way of assessing sense of humor, and the coefficients might have been slightly affected by the rather low reliability of the enjoyment of humor subscale, one might argue that a measurement which is closer to the interventions and more sensitive for (upward) changes, would be able to detect moderating qualities of sense of humor; in this case we would argue similarly to what Seligman et al. (2005) have put forward when introducing the Authentic Happiness Inventory for the assessment of happiness in PPI studies (Proyer et al., in press). However, our findings show that changes in sense of humor are associated with success in the interventions. The changes in sense of humor from pretest to 1 month after the intervention predicted the changes in happiness and depressive symptoms for up to 6 months. Thus, sense of humor might be a working mechanism for humor-based PPIs. Additionally, other models have recently been put forward, which might also be used for developing interventions, and/or assessing the moderating role of humor-related variables (see Ruch, 2012; Ruch and Heintz, 2016; Ruch et al., 2018a).

It is argued that humor-based interventions have a great potential for improving well-being. Given that there is large variety in how humorous behavior is expressed in daily life (Craik et al., 1996; Heintz, 2017) it would be interesting to study (a) whether certain of these behaviors are more strongly related to changes in the desired direction than others and (b) whether personality and/or sense of humor moderate effects of interventions that are based on different humorous behaviors (e.g., those pursued alone vs. in groups). For the latter, a new theoretical framework is needed that enables differentiating among types of humor. One such framework could be the study of the shared and distinct effects of interventions based on preferences and usages of comic styles (i.e., fun, humor, nonsense, wit, irony, satire, sarcasm, and cynicism; Ruch et al., 2018a). Such an approach will help developing humor-based interventions and the study of potentially moderating effects further in a structured way.

### Limitations

We do not know what exactly the participants in our study wrote down and what they experienced as being funny. The latter is a limitation of online studies in general as it is more difficult to control whether and to which degree (as instructed, more or less) participants completed the assigned interventions at home (or in other environments) on their own (or with help of others). This is of particular importance as it was shown that such factors (i.e., continued practice of an intervention, effort invested in the activity, the preference for an activity, or early reactivity in the desired direction) are potent predictors of the effectiveness of an intervention (Schueller, 2010; Proyer et al., 2015; see also Lyubomirsky and Layous, 2013).

Another limitation of Study 1 is, that the sample consisted solely of women. This was due to the opportunity to advertise the study through an article in a women’s magazine. Thus, we do not know, if extraversion would also moderate the effectiveness of humor-based PPIs in men, or if other basic personality traits would play a role in a more diverse sample. Hence, a replication with a more diverse (also in terms of ages represented) and representative sample will be needed for strengthening the findings and exploring potential further moderation effects.

A limitation of both studies is that the dependent variables and also the potential moderators were assessed via self-reports only. Thus, it would be helpful to have more objective indicators of these variables (e.g., including peer-ratings from knowledgeable others). To the best of our knowledge there is only one study that has also considered peer-reports in a humor-based intervention study (or intervention study in general; Ruch et al., 2018b). One might argue that sense of humor is a highly observable trait and, thus, people might also react differently if a person behaves more humorously after an intervention. Their feedback (e.g., eliciting positive emotions due to joint laughter; verbal and facial reactions; etc.) may encourage future humorous behavior, which may have a further positive effect. Hence, it could be tested whether perceived changes in sense of humor by others are associated with changes in the dependent variables. It would also be interesting to see whether an inept use of humor would leads to more negative feedbacks and may even have detrimental effects. One might think of, for example, gelotophobes that have difficulties seeing positive effects in humor and may experience or only anticipate laughter in others as being negative or feel uncomfortable when trying to engage more actively with humor (for an overview see Ruch et al., 2014). Additionally, we did not have data available for sense of humor (in McGhee’s conceptualization) *and* the basic personality traits simultaneously for a joint analysis. Thus, the present findings warrant more investigations of potential moderators of humor-based PPIs, for example to examine their relative importance.

## Ethics Statement

The federal ethics committee of the canton of Zurich, Switzerland provided approval. These studies were carried out in accordance with the recommendations of the Ethical Principles of Psychologists and Code of Conduct (APA) and the Ethical Guidelines for Psychologists of the Swiss Psychological Society (SGP), as outlined by the ethics committee of the Faculty of Arts at the University of Zurich, with online informed consent from all subjects. All subjects gave online informed consent in accordance with the Declaration of Helsinki.

## Author Contributions

SW, RP, and WR: conception or design of the work, data collection, data analysis and interpretation, and final approval of the published version. SW: drafted the article. RP and WR: critical revision of the article.

## Conflict of Interest Statement

The authors declare that the research was conducted in the absence of any commercial or financial relationships that could be construed as a potential conflict of interest.

## Footnotes

1 The attrition rate reported there was 37% in the intervention group and 51% in the placebo control group.
